# Lipid Subclasses Differentiate Insulin Resistance by Triglyceride–Glucose Index

**DOI:** 10.3390/metabo15050342

**Published:** 2025-05-20

**Authors:** Khaled Naja, Najeha Anwardeen, Omar Albagha, Mohamed A. Elrayess

**Affiliations:** 1 Biomedical Research Center, QU Health, Qatar University, Doha P.O. Box 2713, Qatar; khaled.naja@qu.edu.qa (K.N.); n.anwardeen@qu.edu.qa (N.A.); 2 Division of Genomics and Translational Biomedicine, College of Health and Life Sciences, Hamad Bin Khalifa University (HBKU), Doha P.O. Box 34110, Qatar; oalbagha@hbku.edu.qa; 3 Qatar Biomedical Research Institute (QBRI), Hamad Bin Khalifa University, Doha P.O. Box 34110, Qatar; 4 College of Medicine, QU Health, Qatar University, Doha P.O Box 2713, Qatar

**Keywords:** metabolomics, plasmalogens, sphingomyelins, glycerophospholipids

## Abstract

Background: Insulin resistance is a key driver of metabolic syndrome and related disorders, yet its underlying metabolic alterations remain incompletely understood. The Triglyceride–Glucose (TyG) index is an emerging, accessible marker for insulin resistance, with growing evidence supporting its clinical utility. This study aimed to characterize the metabolic profiles associated with insulin resistance using the TyG index in a large, population-based cohort, and to identify metabolic pathways potentially implicated in insulin resistance. Methods: Here, we conducted a cross-sectional study using data from the Qatar Biobank, including 1255 participants without diabetes classified as insulin-sensitive or insulin-resistant based on TyG index tertiles. Untargeted serum metabolomics profiling was performed using high-resolution mass spectrometry. Our statistical analyses included orthogonal partial least squares discriminate analysis and linear models. Results: Distinct metabolic signatures differentiated insulin-resistant from insulin-sensitive participants. Phosphatidylethanolamines, phosphatidylinositols, and phosphatidylcholines, were strongly associated with insulin resistance, while plasmalogens and sphingomyelins were consistently linked to insulin sensitivity. Conclusions: Lipid-centric pathways emerge as potential biomarkers and therapeutic targets for the early detection and personalized management of insulin resistance and related metabolic disorders. Longitudinal studies are warranted to validate causal relationships.

## 1. Introduction

Insulin resistance (IR) is a metabolic condition characterized by the reduced ability of insulin to perform its role in glucose uptake and utilization [1]. IR is driven by multifactorial mechanisms, including ectopic lipid accumulation, chronic inflammation, and endoplasmic reticulum stress, which collectively impair insulin signaling pathways in key tissues such as the liver, skeletal muscle, and adipose tissue [2]. If unmanaged, IR can progress into type 2 diabetes (T2D), cardiovascular diseases, and other severe health issues [3]. These outcomes are linked to the broader effects of IR on lipid metabolism, inflammation, and energy homeostasis, making it a central driver of metabolic syndrome and a significant public health concern. IR is becoming increasingly prevalent worldwide, with recent studies estimating its global prevalence to range from 15.5% to 46.5% [4,5,6], and certain regions exhibiting notably higher rates [7].

The hyperinsulinemic euglycemic clamp (HEC) remains the gold standard for assessing insulin resistance; however, it is too complex for large-scale studies [8]. Clinical alternatives include validated tools such as the homeostatic model assessment for insulin resistance (HOMA-IR), and the Triglyceride–Glucose (TyG) index. The TyG index is emerging as a robust and accessible tool for IR assessment, with growing evidence supporting its role in predicting diabetes, cardiovascular risk, and metabolic complications [9]. The TyG index exhibits a comparable or superior predictive efficacy to HOMA-IR [10,11], with studies showing an excellent association with HEC method in measuring insulin sensitivity [12].

Metabolomics has emerged as a powerful tool for understanding IR and its progression to T2D. Distinct metabolic signatures, including altered levels of steroids, long-chain fatty acids, microbiota byproducts, and amino acids have been identified as biomarkers for insulin resistance [13,14]. Despite extensive research, the precise metabolic alterations and pathways associated with IR remain partially understood. Our objective is to conduct a comprehensive comparison of the metabolic profiles of individuals with insulin resistance and their insulin-sensitive counterparts using the TyG index as a reliable marker to classify insulin resistance status. Additionally, we aim to identify and analyze the metabolic pathways associated with these profiles to better understand the biochemical mechanisms underlying IR. Examining these key metabolites and pathway alterations will help us uncover potential biomarkers and therapeutic targets that could contribute to early detection, risk stratification, and personalized treatment strategies for IR and related metabolic disorders.

## 2. Methods

### 2.1. Data Source and Study Participants

This study utilized data from the Qatar Biobank (QBB), which included detailed information on Qatari nationals and long-term residents [15]. The data collection included a comprehensive socio-demographic questionnaire and various clinical parameters [16]. All these measurements were performed at the Hamad Medical Corporation’s central laboratory, which is certified by the College of American Pathologists. Additionally, the dataset included information on medication usage [17], medical history of diabetes, and a metabolomics profile covering more than 1000 metabolites using the Metabolon platform [18]. This research was approved by the Qatar Biobank’s Institutional Review Boards (QF-QBB-RES-ACC-00178).

Triglyceride–Glucose (TyG) index values were calculated for all individuals using the formula:

$$\mathrm{TyG}\;\mathrm{index}=\mathrm{Ln}\;(\frac{\mathrm{Triglyceride}\;\left(\mathrm{in}\frac{\mathrm{mg}}{\mathrm{dL}}\right)\times \mathrm{Glucose}\;\left(\mathrm{in}\frac{\mathrm{mg}}{\mathrm{dL}}\right)}{2})$$

From an initial cohort of 2998 participants, individuals with T2D were excluded through self-reporting and clinical data, resulting in 2350 participants without diabetes. These participants were then stratified into tertiles according to their TyG index, with the middle tertile excluded to maximize contrast between metabolic profiles and address challenges in defining universal cut-off values across ethnicities and populations [8,19]. This approach retained 1872 participants in the lowest (insulin-sensitive) and highest (insulin-resistant) tertiles for subsequent analysis. To further reduce bias and ensure covariate balance, propensity score matching was performed using the MatchIt package in R (V. 4.2.1), adjusting for age, sex, and BMI. Nearest-neighbor matching yielded a final cohort of 1255 participants.

### 2.2. Metabolomics

All participant serum samples were subjected to untargeted metabolomics using established protocols by Metabolon [20]. Metabolite measurement was performed using a Thermo Scientific Q-Exactive high-resolution/accurate mass spectrometer (Thermo Fisher Scientific, Inc., Waltham, MA, USA) interfaced with a heated electrospray ionization (HESI-II) source and an Orbitrap mass analyzer operated at 35,000 mass resolution, along with Waters ACQUITY ultra-performance liquid chromatography (UPLC) (Waters Corporation, Milford, MA, USA). A thorough explanation of the process has already been provided [20]. Hits were matched with pre-existing library entries of over 3300 pure standard chemicals to identify the compounds. The compounds were divided into several groups according to their sources. Internal standards and quality checks have been previously published [21]. In short, to adjust for discrepancies in sample preparation and instrument performance, a combination of stable isotope-labeled chemicals was utilized for our internal standards. The stability and repeatability of the procedure were tracked over time using quality control samples. To reduce variability and guarantee the integrity of the samples, a systematic methodology was employed for pre-analytical sample management, including sample collection, storage, and preparation.

### 2.3. Statistical Analysis

Principal Component Analysis (PCA) was conducted on the metabolomics data to assess data quality and variation. Orthogonal partial least squares discriminant analysis (OPLS-DA) was performed to differentiate the metabolic signature associated with insulin-sensitive and insulin-resistant groups using SIMCA version 18. For each metabolite, a linear regression model was applied with metabolite level as the dependent variable and the insulin sensitivity group as the main independent variable. Age, sex, BMI, fasting time, and the first two principal components (PC1 and PC2) from the PCA were included as covariates to adjust for potential confounding. To interpret the biological relevance of the metabolomic changes, functional enrichment analysis was conducted. Metabolites with a False Discovery Rate (FDR) < 0.0001 were selected, and the Wilcoxon rank-sum test was used to assess the enrichment of the metabolic pathways. Enriched pathways were identified based on the over-representation of significantly altered metabolites. Associations between the significant metabolites and clinical parameters were analyzed across the full cohort using Spearman’s correlation test. All statistical analysis and figures were produced using R (4.2.1).

## 3. Results

### 3.1. General Characteristics of Participants

We employed propensity score matching techniques to balance the demographic characteristics of age, BMI, and sex between the studied groups. As a result of this procedure, the final sample consisted of 1255 participants. Despite the application of propensity matching to balance age, sex, and BMI between groups, Table 1 shows that age, BMI, and other clinical parameters remained significantly different between the study groups. However, the difference was notably reduced compared to the unmatched dataset.

### 3.2. Multivariate Analysis

**Figure 1:**
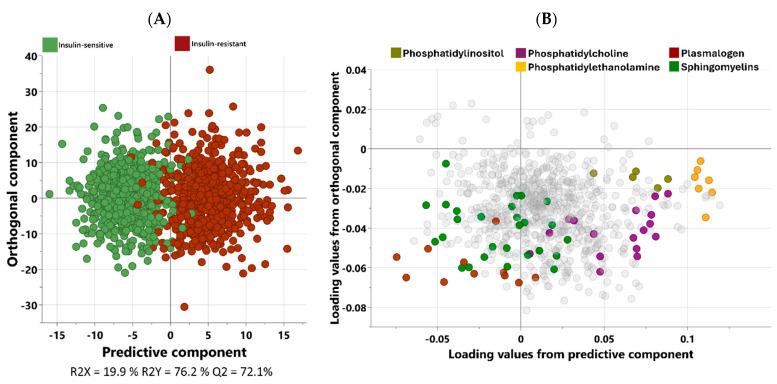
The OPLS-DA score (**A**) and loading plots (**B**) between the insulin-resistant (IS) and insulin-sensitive (TyG-low) groups across all participants (*n* = 1255). R2Y = 76.2%; Q2 = 72.1%. The metabolites in color are the key discriminators, while the less influential metabolites are shown in gray to reduce visual noise.

Non-targeted metabolomics analysis was performed to investigate the metabolic signature of each group. Orthogonal partial least squares discriminant analysis (OPLS-DA) was used to identify the best distinguishing components between the two groups, as shown in Figure 1. The scatter plot in Figure 1A clearly exhibits the distinct separation of the two groups. Figure 1B displays the corresponding loading plots, revealing the primary metabolites responsible for distinguishing the two study groups.

### 3.3. Univariate Analysis

Linear regression analysis was performed to determine the metabolites differentiating between the two groups. The model also contained age, sex, BMI, fasting time, and principal components 1 and 2 from the PCA. The results showed many significant differences in FDR between the two studied groups. Table 2 shows the most FDR-significant metabolites. Phosphatidylethanolamines (PEs), phosphatidylinositols (PIs), and phosphatidylcholines (PCs) exhibited strong correlations with insulin resistance. In contrast, all plasmalogens and sphingomyelins consistently demonstrated significant positive associations with insulin sensitivity. Additionally, sex-specific analyses were conducted for both men and women, yielding similar results (see Supplementary Tables S1 and S2).

### 3.4. Functional Enrichment Analysis

**Figure 2:**
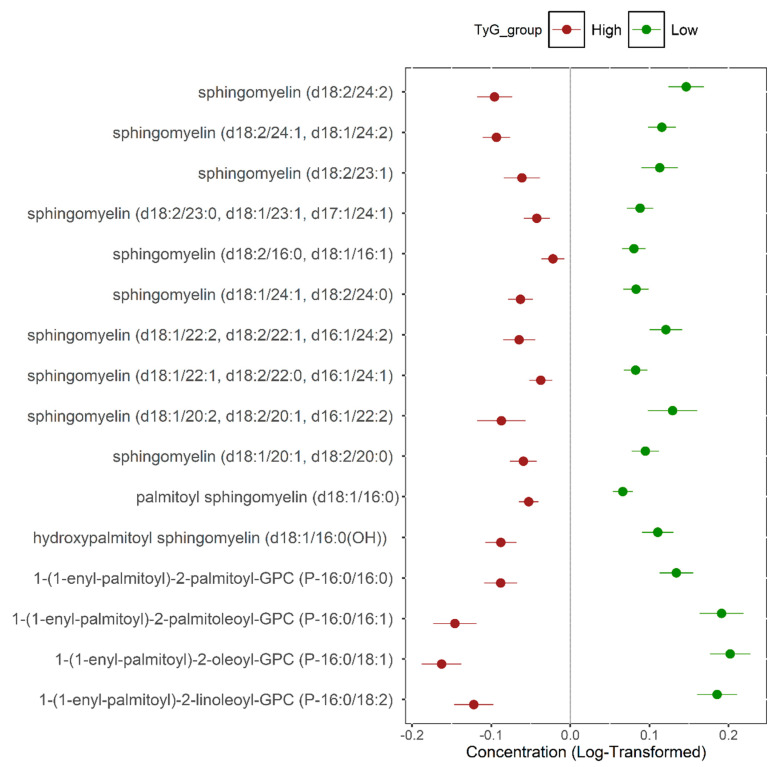
The metabolites significantly associated with insulin sensitivity (FDR < 0.001) from the enriched pathways. The dots and bars represent the means and the 95% confidence intervals of the normalized metabolite values.

The results of the functional enrichment analysis (Table 3) indicated significant differences in the pathways of sphingomyelins, plasmalogens, phosphatidylethanolamines, phosphatidylcholines, and phosphatidylinositols. Figure 2 shows the metabolites significantly (FDR < 0.001) associated with insulin sensitivity (sphingomyelins and plasmalogens) from the enriched pathways.

### 3.5. Association Between Metabolites Associated with Insulin Sensitivity and Clinical Traits

**Figure 3:**
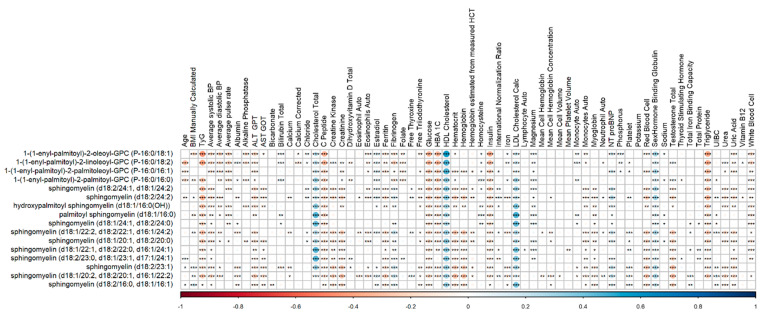
A correlation heatmap showing Spearman’s correlation between the significant metabolites associated with insulin sensitivity and clinical parameters. The size of the circles within each cell corresponds to the magnitude of Spearman’s correlation coefficient. The color intensity in each cell represents the strength and direction of the correlation between a specific metabolite and a clinical trait. (*/**/*** denote a *p*-value <0.05/<0.01/<0.001).

Spearman’s correlation demonstrated a strong positive association between sphingomyelins and plasmalogens with HDL-cholesterol. Conversely, they exhibited negative correlations with glucose, triglycerides, C-peptide, and insulin. To promote clarity and reduce complexity, only the metabolites associated with insulin sensitivity are presented (Figure 3).

## 4. Discussion

Metabolomics offers a powerful platform for advancing our understanding of insulin resistance by identifying biomarkers, elucidating disease mechanisms, and supporting the development of targeted interventions. The TyG index is a reliable biomarker for assessing IR and related metabolic disorders. In this study, we compared the metabolic profiles of insulin-resistant and insulin-sensitive individuals using the TyG index for IR classification.

Our results revealed a clear dichotomy in lipid subclass associations with insulin resistance (high TyG) and sensitivity (low TyG), suggesting differential metabolic profiles tied to insulin resistance and cardiometabolic risk, and emphasizing the importance of lipid structure and metabolism in glucose homeostasis. Interestingly, our results showed that all phosphatidylethanolamines (PEs), phosphatidylinositols (PIs), and phosphatidylcholines (PCs) exhibited strong correlations with insulin resistance. In contrast, all plasmalogens and sphingomyelins consistently demonstrated significant positive associations with insulin sensitivity.

PE, PI, and PC are glycerophospholipids composed of a glycerol backbone with two hydrophobic fatty acid tails and a hydrophilic phosphate group linked to different head groups: ethanolamine, inositol, or choline, respectively. These glycerophospholipids are essential components of cellular membranes and play key roles in maintaining membrane fluidity, vesicle trafficking, and cellular signaling [22].

While these glycerophospholipids are critical contributors to metabolic regulation, their exact roles in insulin resistance remain a subject of debate [23]. Plasma PE and PC levels have been shown to be associated with insulin resistance in population studies [24,25]. Our previous study revealed increased levels of these glycerophospholipids in participants with IS and T2D [26]. Relatedly, Razquin et al. reported, in a case–cohort study, that baseline PE levels were positively associated with T2D risk [27]. Additionally, Yang et al. [28] observed significant increases in PE (22:6/16:0) and PC (16:0/20:4) levels in prediabetes and T2D patients. Notably, PC and PE are key components of VLDL, which transports triglycerides, and increased VLDL secretion is a hallmark of IR-associated dyslipidemia [29]. However, Zhao et al. [30] showed that phosphatidylcholine (22:6, 20:4) was significantly associated with a decreased risk of diabetes. Experimental findings have also produced conflicting results, highlighting the complexity of their involvement. For instance, in a mouse model, experimentally reduced phosphatidylcholine levels were shown to lead to triacylglycerol accumulation but had no effect on insulin sensitivity [31]. However, decreased phosphatidylcholine synthesis resulted in improved insulin sensitivity in mice treated with a high-fat diet [32].

In our study, the elevated plasma levels of PC, PE, and PI observed in insulin-resistant individuals may result from a complex interplay of metabolic disturbances, such as increased lipoprotein production and altered membrane lipid remodeling. These alterations in the levels or composition of these phospholipids can disrupt membrane organization and impair insulin receptor localization and signaling efficiency, ultimately leading to reduced insulin sensitivity. It is patently clear that these heightened phospholipid concentrations indicate a disruption in phospholipid metabolism that is closely associated with insulin resistance. Future studies should explore the molecular mechanisms underlying these observed alterations.

Sphingomyelins and plasmalogens are two distinct classes of membrane lipids with important structural and functional roles. Sphingomyelins are sphingophospholipids which consist of a ceramide backbone (sphingosine plus a fatty acid) linked to a phosphocholine or phosphoethanolamine head group. Plasmalogens are a unique subclass of glycerophospholipids characterized by a vinyl–ether bond at the sn-1 position of the glycerol backbone and an ester bond at the sn-2 position.

Sphingomyelins are critical constituents of lipid rafts, specialized membrane microdomains that house insulin receptors and signaling proteins [33]. The dysregulation of sphingolipid metabolism correlates with the pathogenesis of metabolic diseases in humans [34]. Griess et al. [35] showed that sphingomyelins are essential for proper proinsulin processing and systemic glucose homeostasis through their role in maintaining β-cell functionality. Moreover, Straczkowski [36] et al. showed that the sphingomyelin signaling pathway in muscle is an important factor determining the development of IR. In terms of acyl chain length and saturation levels, studies have reported differing associations between sphingomyelin species and insulin resistance. Semba et al. [37] found that sphingomyelin species like C16:0, C24:1, and C26:1 were linked to lower odds of abnormal HOMA-IR results in older adults. However, some experimental studies have suggested that decreasing sphingomyelin may be associated with increasing insulin sensitivity [23]. For instance, Hanamatsu et al. [38] demonstrated that the levels of SM species containing saturated acyl chains (C18:0, C20:0, C22:0, and C24:0) positively correlated with the parameters of insulin resistance.

Higher sphingomyelins may also reflect a shift away from ceramide accumulation, thus promoting insulin sensitivity. Ceramides are central mediators in the development of insulin resistance, acting through multiple molecular pathways to disrupt insulin signaling, promote inflammation, and alter lipid metabolism [39,40]. Our insulin-sensitive group might have a healthier sphingolipid profile, with efficient sphingomyelin synthesis and/or reduced conversion to ceramides. Concordantly, Bandet et al. [41] showed that the overexpression of the muscle-specific ceramide transporter, which diverts ceramides for sphingomyelin synthesis, enhanced insulin sensitivity in mice. Additionally, Kolak et al. [42] revealed that the expression of the major human neutral sphingomyelinase, the enzyme that hydrolyzes sphingomyelin to generate ceramide, was elevated in inflamed adipose tissue compared to healthy tissue in obese women. Interestingly, Zemski et al. showed that circulating oxidized phosphatidylcholine induces ceramide accumulation, reduces sphingomyelins, triggers inflammatory signaling, and causes insulin resistance in muscle [43].

Plasmalogens are phospholipids with antioxidant properties that protect cellular membranes from oxidative damage [44]. Plasmalogens may enhance membrane fluidity and receptor function, thereby improving insulin signaling [45]. Our findings align with emerging evidence that plasmalogen deficiency is linked to metabolic disorders, including T2D [46]. For instance, Razquin et al. showed that plasmalogens, sphingomyelins, and cholesterol esters were inversely associated with the risk of T2D [27]. Additionally, Zhang et al. [47] reported that patients with polycystic ovary syndrome and insulin resistance have significantly lower levels of plasmalogens in their follicular fluid compared to controls. While Tonks et al. [48] reported that lower plasma plasmalogen levels were associated with obesity but not with insulin resistance after adjusting for adiposity, our findings suggest that higher plasmalogen levels are linked to insulin sensitivity after correcting for BMI. It is important to acknowledge, however, that the study by Tonks et al. [48] was conducted with a relatively modest sample size, which may have influenced the observed associations. The positive correlation of plasmalogens with the insulin-sensitive group in our study suggests their protective role in maintaining metabolic health. These observations are further corroborated by our correlation analysis. Both sphingomyelins and plasmalogens consistently demonstrate strong positive associations with HDL-cholesterol while exhibiting significant negative correlations with glycemic markers (glucose, HbA1c, C-peptide, and insulin) and triglycerides. Enhancing plasmalogen synthesis or supplementation could represent a novel therapeutic strategy to improve insulin sensitivity.

While our study identifies robust associations between phospholipid profiles and insulin resistance, several limitations necessitate cautious interpretation. The cross-sectional design precludes causal inference, leaving unresolved whether the observed lipid changes drive IR or result from it. However, the large sample size and robust statistical approach strengthen the reliability of our observed associations. Additionally, the absence of dietary profiling and gut microbiome data represents another limitation, as both factors can influence lipid metabolism and insulin resistance. Furthermore, the residual confounding of age differences between our studied groups cannot be entirely excluded; however, the convergence of the matched cohort design, covariate adjustment, and biological plausibility strongly supports the internal validity of the reported metabolic signatures. This is also supported by the consistency of the lipid subclass associations with prior longitudinal studies, suggesting that our age adjustments effectively captured true biological relationships rather than demographic artifacts.

## 5. Conclusions

This study reveals distinct phospholipid profiles linked to insulin resistance and sensitivity, with glycerophospholipids strongly associated with insulin resistance and sphingomyelins and plasmalogens correlated with insulin sensitivity. The TyG index effectively stratifies these metabolic patterns, highlighting lipid structure and metabolism as central to glucose dysregulation. Elevated PC, PE, and PI levels in insulin-resistant individuals likely reflect disrupted phospholipid metabolism and increased VLDL production, while higher sphingomyelin levels may indicate reduced ceramide accumulation and improved insulin signaling. Plasmalogens’ antioxidant properties and membrane-stabilizing roles further support their protective association with metabolic health.

These findings highlight the value of lipidomics in identifying robust metabolic signatures of insulin resistance, with specific phospholipids emerging as strong biomarkers. Given the global burden of metabolic syndrome, our results suggest that lipid-centric pathways may serve as universal targets for early detection and intervention across diverse populations. Longitudinal and mechanistic studies are needed to determine if these lipid profiles precede insulin resistance or result from compensatory mechanisms. Future research should also validate these signatures in other populations and integrate multi-omics and lifestyle data to advance precision medicine for metabolic disorders.

## Figures and Tables

**Table 1:** Demographic characteristics of participants.

Test	Variable	Insulin-Sensitive Group (N = 620)	Insulin-Resistant Group (N = 635)	*p*-Value
General characteristics	Sex (M/F)	322/298	349/286	0.3
	Age	35 (29–43.25)	47 (41–54.5)	<0.0001
	BMI (kg/m^2^)	27.74 (24.7–31.76)	30.48 (27.26–34.12)	<0.0001
	Waist to hip ratio	0.8 (0.75–0.86)	0.9 (0.83–0.96)	<0.0001
	Systolic blood pressure (mmHg)	109 (102–118)	121 (112–131)	<0.0001
	Diastolic blood pressure (mmHg)	71 (66–77.5)	78 (72–85)	<0.0001
Insulin resistance	TyG index	8 (7.83–8.16)	9.21 (9.01–9.52)	<0.0001
Blood sugar	Fasting blood glucose (mmol/L)	4.9 (4.6–5.16)	5.9 (5.1–8.1)	<0.0001
	HbA_1C_ (%)	5.3 (5.1–5.6)	5.9 (5.5–6.8)	<0.0001
	C-peptide (ng/mL)	1.88 (1.39–2.52)	3.39 (2.49–4.84)	<0.0001
	Insulin (uU/mL)	7.2 (5–11)	17 (10.1–30)	<0.0001
Lipid profile	Total cholesterol (mmol/L)	4.6 (4.14–5.2)	5.23 (4.56–5.9)	<0.0001
	HDL-cholesterol (mmol/L)	1.46 (1.23–1.73)	1.11 (0.96–1.31)	<0.0001
	LDL-cholesterol (mmol/L)	2.9 (2.19–3.24)	3 (2.46–3.83)	<0.0001
	Triglyceride (mmol/L)	0.77 (0.63–0.9)	2.04 (1.68–2.6)	<0.0001
Cardiac function	NT-proBNP (pg/mL)	27 (14.78–44)	20.6 (11.83–38.15)	0.0003
	Homocysteine (µmol/L)	8.4 (7–10.25)	8.3 (6.8–10.1)	0.3261
Kidney function	Chloride (mmol/L)	101 (100–103)	101 (99–102)	<0.0001
	Urea (mmol/L)	4.2 (3.5–5)	4.5 (3.7–5.3)	0.0001
	Bicarbonate (mmol/L)	27 (25–28)	27 (25–28)	0.6
	Total protein (g/L)	73 (70–75)	73 (70–75)	0.7
Liver function	Albumin (g/L)	45 (43–47)	45 (43–47)	0.0034
	Bilirubin (µmol/L)	6.6 (5–9)	5.9 (4–8)	<0.0001
	ALT (U/L)	16 (12–23)	22 (16–32)	<0.0001
	AST (U/L)	17 (14–21)	18 (15–23)	0.0092

The clinical parameters were checked for Gaussian distribution using the Shapiro–Wilk test. The data were then presented as median (IQR) or mean (SD). The medians/means between the study groups were compared using the Mann–Whitney/Student *t* tests. A *p*-value of <0.05 was considered significant. Abbreviations: BMI, body mass index; HbA1C, glycated hemoglobin; HDL, high-density lipoprotein; LDL, low-density lipoprotein; NT-proBNP, N-terminal pro–B-type natriuretic peptide; ALT, alanine transaminase; AST, aspartate aminotransferase.

**Table 2:** Linear regression analysis to determine top metabolites associated with insulin resistance, while adjusting for age, sex, BMI, and principal components 1 and 2.

Metabolite	Superpathway	Subpathway	Estimate	SE	*p*-Value	FDR
1-palmitoyl-2-oleoyl-GPE (16:0/18:1)	Lipid	Phosphatidylethanolamine	−0.72	0.033	5.5 × 10^−84^	1.6 × 10^−81^
1-palmitoyl-2-arachidonoyl-GPE (16:0/20:4)	Lipid	Phosphatidylethanolamine	−0.57	0.026	7.2 × 10^−82^	1.6 × 10^−79^
1-palmitoyl-2-linoleoyl-GPE (16:0/18:2)	Lipid	Phosphatidylethanolamine	−0.66	0.031	3.6 × 10^−77^	5.2 × 10^−75^
1-stearoyl-2-linoleoyl-GPE (18:0/18:2)	Lipid	Phosphatidylethanolamine	−0.64	0.030	9.0 × 10^−77^	1.1 × 10^−74^
1-stearoyl-2-oleoyl-GPE (18:0/18:1)	Lipid	Phosphatidylethanolamine	−0.67	0.032	3.0 × 10^−76^	3.3 × 10^−74^
1-stearoyl-2-arachidonoyl-GPE (18:0/20:4)	Lipid	Phosphatidylethanolamine	−0.51	0.026	3.5 × 10^−68^	2.8 × 10^−66^
1-(1-enyl-palmitoyl)-2-oleoyl-GPC (P-16:0/18:1)	Lipid	Plasmalogen	0.36	0.020	1.1 × 10^−59^	7.9 × 10^−58^
1-palmitoyl-2-docosahexaenoyl-GPE (16:0/22:6)	Lipid	Phosphatidylethanolamine	−0.60	0.035	1.1 × 10^−54^	6.8 × 10^−53^
1-(1-enyl-palmitoyl)-2-palmitoleoyl-GPC (P-16:0/16:1)	Lipid	Plasmalogen	0.34	0.021	4.7 × 10^−47^	2.6 × 10^−45^
1-(1-enyl-palmitoyl)-2-linoleoyl-GPC (P-16:0/18:2)	Lipid	Plasmalogen	0.30	0.019	3.2 × 10^−46^	1.7 × 10^−44^
Sphingomyelin (d18:2/24:1, d18:1/24:2)	Lipid	Sphingomyelins	0.21	0.013	5.9 × 10^−46^	2.9 × 10^−44^
Sphingomyelin (d18:2/24:2)	Lipid	Sphingomyelins	0.25	0.017	9.8 × 10^−41^	4.1 × 10^−39^
1-palmitoyl-2-palmitoleoyl-GPC (16:0/16:1)	Lipid	Phosphatidylcholine	−0.39	0.029	6.0 × 10^−36^	2.2 × 10^−34^
1-(1-enyl-palmitoyl)-2-palmitoyl-GPC (P-16:0/16:0)	Lipid	Plasmalogen	0.22	0.016	1.9 × 10^−35^	6.6 × 10^−34^
1-palmitoyl-2-arachidonoyl-GPI (16:0/20:4)	Lipid	Phosphatidylinositol	−0.37	0.028	1.3 × 10^−34^	4.3 × 10^−33^
Hydroxypalmitoyl sphingomyelin (d18:1/16:0(OH))	Lipid	Sphingomyelins	0.20	0.015	2.5 × 10^−32^	7.5 × 10^−31^
1-myristoyl-2-arachidonoyl-GPC (14:0/20:4)	Lipid	Phosphatidylcholine	−0.43	0.036	5.9 × 10^−32^	1.7 × 10^−30^
Sphingomyelin (d18:1/22:2, d18:2/22:1, d16:1/24:2)	Lipid	Sphingomyelins	0.19	0.017	4.7 × 10^−29^	1.3 × 10^−27^
Sphingomyelin (d18:1/20:1, d18:2/20:0)	Lipid	Sphingomyelins	0.16	0.014	4.7 × 10^−28^	1.3 × 10^−27^
Sphingomyelin (d18:1/24:1, d18:2/24:0)	Lipid	Sphingomyelins	0.15	0.013	8.7 × 10^−29^	2.3 × 10^−27^
Palmitoyl sphingomyelin (d18:1/16:0)	Lipid	Sphingomyelins	0.12	0.010	3.0 × 10^−28^	7.7 × 10^−27^
Glucose	Carbohydrate	Glycolysis, Gluconeogenesis, and Pyruvate Metabolism	−0.22	0.020	8.2 × 10^−28^	2.1 × 10^−26^
1-palmitoyl-2-dihomo-linolenoyl-GPC (16:0/20:3n3 or 6)	Lipid	Phosphatidylcholine	−0.28	0.025	1.3 × 10^−27^	3.1 × 10^−26^
1-stearoyl-2-arachidonoyl-GPI (18:0/20:4)	Lipid	Phosphatidylinositol	−0.21	0.019	1.4 × 10^−27^	3.4 × 10^−26^
1-palmitoyl-2-arachidonoyl-GPC (16:0/20:4n6)	Lipid	Phosphatidylcholine	−0.19	0.018	9.9 × 10^−26^	2.1 × 10^−24^
1-palmitoyl-2-oleoyl-GPI (16:0/18:1)	Lipid	Phosphatidylinositol	−0.30	0.029	1.7 × 10^−24^	3.5 × 10^−23^
1-palmitoyl-2-linoleoyl-GPI (16:0/18:2)	Lipid	Phosphatidylinositol	−0.28	0.027	3.0 × 10^−24^	6.0 × 10^−23^
N-Lactoyl phenylalanine	Amino Acid	Phenylalanine Metabolism	−0.28	0.028	7.7 × 10^−23^	1.4 × 10^−21^
Sphingomyelin (d18:1/22:1, d18:2/22:0, d16:1/24:1)	Lipid	Sphingomyelins	0.12	0.012	2.5 × 10^−22^	4.5 × 10^−21^
Sphingomyelin (d18:2/23:1)	Lipid	Sphingomyelins	0.18	0.018	2.8 × 10^−22^	4.8 × 10^−21^
Pyruvate	Carbohydrate	Glycolysis, Gluconeogenesis, and Pyruvate Metabolism	−0.16	0.016	3.3 × 10^−22^	5.5 × 10^−21^
Sphingomyelin (d18:2/23:0, d18:1/23:1, d17:1/24:1)	Lipid	Sphingomyelins	0.13	0.013	3.8 × 10^−22^	6.4 × 10^−21^
1-palmitoyl-2-oleoyl-GPC (16:0/18:1)	Lipid	Phosphatidylcholine	−0.16	0.016	5.2 × 10^−22^	8.5 × 10^−21^
Sphingomyelin (d18:1/20:2, d18:2/20:1, d16:1/22:2)	Lipid	Sphingomyelins	0.22	0.025	4.3 × 10^−18^	5.5 × 10^−17^
1-myristoyl-2-palmitoyl-GPC (14:0/16:0)	Lipid	Phosphatidylcholine	−0.32	0.037	1.7 × 10^−17^	2.1 × 10^−16^
Sphingomyelin (d18:2/16:0, d18:1/16:1)	Lipid	Sphingomyelins	0.10	0.012	2.8 × 10^−17^	3.3 × 10^−16^

**Table 3:** Enriched pathways from functional enrichment analysis using Wilcoxon sum of ranks test.

Enriched Pathways	*p*-Value	FDR
Sphingomyelins	2.8 × 10^−7^	2.8 × 10^−5^
Phosphatidylethanolamine (PE)	3.6 × 10^−6^	1.7 × 10^−4^
Plasmalogen	8.6 × 10^−6^	2.8 × 10^−4^
Phosphatidylcholine (PC)	7.1 × 10^−5^	1.1 × 10^−3^
Phosphatidylinositol (PI)	3.2 × 10^−4^	4.5 × 10^−3^

## Data Availability

The datasets used and/or analyzed during the current study are available from the corresponding author on reasonable request.

## Supplementary Materials

The following supporting information can be downloaded at: https://www.mdpi.com/article/10.3390/metabo15050342/s1, Table S1. Linear regression analysis to determine top metabolites associated with insulin resistance in men, adjusting for age, BMI, and principal components 1 and 2. Table S2. Linear regression analysis to determine top metabolites associated with insulin resistance in women, adjusting for age, BMI, and principal components 1 and 2.
