# Supplementary material for: Lipid Subclasses Differentiate Insulin Resistance by Triglyceride–Glucose Index
Source: Metabolites. 2025 May 20;15(5):342. doi: 10.3390/metabo15050342 (PMC12113954; doi:10.3390/metabo15050342)
Supplement: Supplementary file 1 [file metabolites-15-00342-s001.zip › metabolites-3638502-supplementary.pdf]

Table S1. Linear regression analysis to determine top metabolites associated with insulin resistance in men, adjusting for age, BMI, and principal components 1 and 2.

Metabolite	Superpathway	Subpathway	Estimate	SE	p-value	FDR
1-palmitoyl-2-oleoyl-GPE (16:0/18:1)	Lipid	Phosphatidylethanolamine	-0.7	0.052	8.7×10^{-36}	1.9×10^{-33}
1-stearoyl-2-linoleoyl-GPE (18:0/18:2)*	Lipid	Phosphatidylethanolamine	-0.63	0.047	1.2×10^{-35}	2.2×10^{-33}
1-palmitoyl-2-linoleoyl-GPE (16:0/18:2)	Lipid	Phosphatidylethanolamine	-0.64	0.050	4.7×10^{-33}	5.8×10^{-31}
1-stearoyl-2-arachidonoyl-GPE (18:0/20:4)	Lipid	Phosphatidylethanolamine	-0.52	0.041	2.4×10^{-32}	2.6×10^{-30}
1-stearoyl-2-oleoyl-GPE (18:0/18:1)	Lipid	Phosphatidylethanolamine	-0.64	0.051	4.6×10^{-32}	4.4×10^{-30}
1-palmitoyl-2-arachidonoyl-GPE (16:0/20:4)*	Lipid	Phosphatidylethanolamine	-0.51	0.042	2.4×10^{-31}	2.0×10^{-29}
1-(1-enyl-palmitoyl)-2-oleoyl-GPC (P-16:0/18:1)*	Lipid	Plasmalogen	0.38	0.031	4.0×10^{-30}	3.2×10^{-28}
1-(1-enyl-palmitoyl)-2-linoleoyl-GPC (P-16:0/18:2)*	Lipid	Plasmalogen	0.32	0.031	7.3×10^{-23}	4.2×10^{-21}
1-stearoyl-2-arachidonoyl-GPI (18:0/20:4)	Lipid	Phosphatidylinositol	-0.28	0.029	4.4×10^{-20}	2.2×10^{-18}
1-(1-enyl-palmitoyl)-2-palmitoleoyl-GPC (P-16:0/16:1)*	Lipid	Plasmalogen	0.33	0.035	7.1×10^{-20}	3.4×10^{-18}
Sphingomyelin (d18:2/24:1, d18:1/24:2)*	Lipid	Sphingomyelins	0.21	0.022	8.3×10^{-19}	3.7×10^{-17}
1-(1-enyl-palmitoyl)-2-palmitoyl-GPC (P-16:0/16:0)*	Lipid	Plasmalogen	0.24	0.027	2.3×10^{-18}	9.5×10^{-17}
1-palmitoyl-2-arachidonoyl-GPI (16:0/20:4)*	Lipid	Phosphatidylinositol	-0.39	0.045	6.3×10^{-17}	2.3×10^{-15}
1-palmitoyl-2-docosahexaenoyl-GPE (16:0/22:6)*	Lipid	Phosphatidylethanolamine	-0.50	0.058	1.7×10^{-16}	5.9×10^{-15}
Hydroxypalmitoyl sphingomyelin (d18:1/16:0(OH))**	Lipid	Sphingomyelins	0.20	0.026	3.8×10^{-14}	1.2×10^{-12}

Table S2. Linear regression analysis to determine top metabolites associated with insulin resistance in women, adjusting for age, BMI, and principal components 1 and 2.

Metabolite	Superpathway	Subpathway	Estimate	SE	p-value	FDR
1-palmitoyl-2-arachidonoyl-GPE (16:0/20:4)*	Lipid	Phosphatidylethanolamine	-0.60	0.038	1.4×10^{-45}	6.3×10^{-43}
1-palmitoyl-2-oleoyl-GPE (16:0/18:1)	Lipid	Phosphatidylethanolamine	-0.70	0.047	1.2×10^{-41}	2.2×10^{-39}
1-palmitoyl-2-linoleoyl-GPE (16:0/18:2)	Lipid	Phosphatidylethanolamine	-0.63	0.044	2.2×10^{-39}	3.1×10^{-37}
1-(1-enyl-palmitoyl)-2-oleoyl-GPC (P-16:0/18:1)*	Lipid	Plasmalogen	0.39	0.027	1.2×10^{-38}	1.5×10^{-36}
1-stearoyl-2-linoleoyl-GPE (18:0/18:2)*	Lipid	Phosphatidylethanolamine	-0.59	0.042	1.9×10^{-37}	2.0×10^{-35}
1-stearoyl-2-oleoyl-GPE (18:0/18:1)	Lipid	Phosphatidylethanolamine	-0.61	0.045	4.9×10^{-36}	4.7×10^{-34}
1-stearoyl-2-arachidonoyl-GPE (18:0/20:4)	Lipid	Phosphatidylethanolamine	-0.51	0.038	9.2×10^{-36}	8.0×10^{-34}
1-palmitoyl-2-docosahexaenoyl-GPE (16:0/22:6)*	Lipid	Phosphatidylethanolamine	-0.65	0.048	1.5×10^{-35}	1.2×10^{-33}
1-(1-enyl-palmitoyl)-2-palmitoleoyl-GPC (P-16:0/16:1)*	Lipid	Plasmalogen	0.36	0.029	2.7×10^{-30}	1.6×10^{-28}
Sphingomyelin (d18:2/24:1, d18:1/24:2)*	Lipid	Sphingomyelins	0.23	0.019	4.4×10^{-30}	2.4×10^{-28}
1-(1-enyl-palmitoyl)-2-linoleoyl-GPC (P-16:0/18:2)*	Lipid	Plasmalogen	0.33	0.028	1.4×10^{-29}	7.0×10^{-28}
Sphingomyelin (d18:2/24:2)*	Lipid	Sphingomyelins	0.27	0.025	1.6×10^{-25}	7.0×10^{-24}
Glucose	Carbohydrate	Glycolysis, Gluconeogenesis, and Pyruvate Metabolism	-0.27	0.027	1.3×10^{-21}	4.8×10^{-20}
1-palmitoyl-2-palmitoleoyl-GPC (16:0/16:1)*	Lipid	Phosphatidylcholine	-0.40	0.040	1.4×10^{-21}	4.9×10^{-20}
Hydroxypalmitoyl sphingomyelin (d18:1/16:0(OH))**	Lipid	Sphingomyelins	0.21	0.020	1.5×10^{-21}	5.3×10^{-20}
